# Bridging the Gap between Ophthalmology and Emergency Medicine in Community-Based Emergency Departments (EDs): A Neuro-Ophthalmology Guide for ED Practitioners

**DOI:** 10.3390/clinpract11040106

**Published:** 2021-12-02

**Authors:** Kristina Thomas, Cindy Ocran, Anna Monterastelli, Alfredo A. Sadun, Kimberly P. Cockerham

**Affiliations:** 1 Department of Emergency Medicine, St. Joseph’s Medical Center, Stockton, CA 95204, USA; kristina.thomas@commonspirit.org; 2 Department of Ophthalmology, Central Valley Eye Medical Group, Stockton, CA 95207, USA; cindyocran@gmail.com; 3 Loyola Marymount University, Los Angeles, CA 90045, USA; annanm83@gmail.com; 4 Doheny Eye Institute, UCLA, Los Angeles, CA 91105, USA; alfredo.sadun@gmail.com; 5 Byers Eye Institute, Department of Ophthalmology, Stanford School of Medicine, Palo Alto, CA 94303, USA

**Keywords:** visual loss, emergency medicine, optic neuritis, neuromyelitis optica, ischemic optic neuropathy, giant cell arteritis, stroke, pituitary apoplexy, aneurysm, cranial nerve palsies, cavernous sinus fistula, cavernous sinus thrombosis, orbital apex syndrome

## Abstract

Coordination of care for patients with neuro-ophthalmic disorders can be very challenging in the community emergency department (ED) setting. Unlike university- or tertiary hospital-based EDs, the general ophthalmologist is often not as familiar with neuro-ophthalmology and the examination of neuro-ophthalmology patients in the acute ED setting. Embracing image capturing of the fundus, using a non-mydriatic camera, may be a game-changer for communication between ED physicians, ophthalmologists, and tele-neurologists. Patient care decisions can now be made with photographic documentation that is then conveyed through HIPAA-compliant messaging with accurate and useful information with both ease and convenience. Likewise, external photos of the anterior segment and motility are also helpful. Finally, establishing clinical and imaging guidelines for common neuro-ophthalmic disorders can help facilitate complete and appropriate evaluation and treatment.

## 1. Introduction

As physicians, we are most comfortable in clinical situations that focus on our own scope of practice. However, we may be faced with medical conditions that we do not routinely manage. This may result in a gap in our knowledge and/or skill set that makes us apprehensive. This problem is well highlighted in the case of patients presenting to a community-based ED with neuro-ophthalmic complaints. Such cases can often fall outside of the comfort zone for all physicians involved, including the ophthalmologist, ED physician, neurologist, and radiologist. The goal of this article is to bring awareness to this gap and provide guidelines and recommendations to increase confidence in physicians as it pertains to common neuro-ophthalmic complaints. In doing so, we hope to optimize the quality of care for the patient. We believe that image acquisition and communication via HIPAA-compliant texts are key to enhancing the quality of care.

Ophthalmology is often not a required clinical rotation in medical school, leaving physicians with minimal exposure to the specialty [1,2]. In 2014, only 18% of medical schools required a clinical ophthalmology rotation in the United States [2]. Most ophthalmologists often have clinical practices that are limited to cataract surgery and common ophthalmic diagnoses, such as dry eyes, glaucoma, diabetes, and macular degeneration. Keeping current with neuro-ophthalmic diagnosis and management is not something the average ophthalmologist can readily accomplish. Thus, many community-based hospitals have little or no ophthalmic coverage, leaving ED physicians unable to obtain adequate consultation. In cases where there is a call group available to the ED, their scope of knowledge regarding complex orbit and neuro-ophthalmology can potentially be limited.

Another confounding issue is that tele-neurology has grown quickly and often times has to make recommendations without visualization of the posterior segment of the eye [3]. Because radiology protocols for neuro-ophthalmic issues are rarely established, the imaging that is obtained can be incorrect or inadequate for accurate diagnosis. For similar reasons, tele-radiology can also have its limitations, with general radiologists misinterpreting critical image findings.

Furthermore, in the authors’ experience, advances in laboratory workup for differential neuro-ophthalmology diagnoses are typically not covered in most ED training programs. This leaves our new generation of emergency physicians with gaps in knowledge as it pertains to the standard of care for this subspeciality practice. This proves more difficult given that neuro-ophthalmic emergencies can be very severe and can cause permanent visual loss and even death. It is crucial for the emergency physician to appropriately work up and manage the neuro-ophthalmic patient during their ED stay.

The purpose of the guide is to bridge the gap between ophthalmologists and emergency physicians. Using a case-based approach, we will describe five common neuro-ophthalmic clinical vignettes that can be encountered based on the authors’ clinical experiences.

## 2. Case 1

A 75-year-old Caucasian woman with a history of well-controlled hypertension and hypercholesterolemia presents to the ED with complaints of a new-onset headache. She describes her headache as constant and refractory to over-the-counter pain relievers. Her physical examination is unremarkable. She has no ocular complaints, and no eye examination is performed. A non-contrast computed tomography (CT) scan of the brain is performed and reported to be normal. She is discharged with a prescription for Vicodin. Two weeks later, she returns to the ED with a worsened headache and blurred vision. The ophthalmologist on call is consulted by telephone. Visual acuity is noted to be 20/25 in both eyes (OU), pupils are round reactive to light, and no afferent pupillary defect is present. The patient has small pupils that precluded an easy view to the back of the eye with a direct ophthalmoscope. Attempts to check intraocular pressure are unsuccessful as the tonometer would not calibrate. A slit lamp examination is not done as the machine is not working. A CT and computed tomography angiogram (CTA) are performed at the recommendation of the tele-neurology doctor on call, both of which are normal. No labs are ordered. The patient is instructed to see the ophthalmologist in the morning. When the patient wakes up the next morning, her vision is worse. On examination in the ophthalmologist’s office, her visual acuity has decreased to 20/400 right eye (OD) and 20/25 left eye (OS).

### Could the Visual Loss Have Been Prevented? What Would Have Been an Optimal Work Up in the Patient?

Giant cell arteritis (GCA) is a common disorder that presents to the ED and should be high on the differential for all elderly patients presenting with a headache, visual loss, or diplopia [4,5]. Table 1 presents the most common presenting symptoms. Asking the right questions is crucial in preventing permanent blindness. On further questioning, the patient denied jaw claudication and temporal tenderness but did complain of ear pain and eye ache. Other historical clues that can be helpful include polymyalgia rheumatica, weight loss, fatigue, and abdominal pain due to mesenteric ischemia [6,7]. Laboratory evaluation should include the erythrocyte sedimentation rate (ESR), C-reactive protein (CRP), and complete blood count including platelet count [8,9]. A list of the most commonly abnormal lab values for the disease process are listed in Table 2. These laboratory tests are elevated at diagnosis in most patients and crucial to monitoring disease activity [8,9]. However, 20% of patients have normal laboratory testing [6]. CRP is much more sensitive than ESR, but the combination of all three is the most helpful and also guides management [6,8,9]. Acute serum amyloid A (A-SAA) is less readily available but also highly sensitive [9]. Magnetic resonance imaging (MRI) with and without gadolinium of the orbits and temporal artery may be very helpful [5,6,8,9]. A CT scan does not pick up the vasculitis but an ultrasound of the temporal arteries may [8]. GCA can cause enhancement of the optic nerve or orbit on the MRI; it also can cause enhancement of the periosteum and temporalis muscle surrounding an occluded or partially occluded temporal artery [7,8]. If there is a history sufficiently suspicious for GCA (even if laboratory tests and imaging normal), the standard of care is to place the patient on 40 mg of prednisone (if no visual symptoms or signs) and refer for a temporal artery biopsy within two weeks [7,8,9]. Rheumatology is then consulted, and the patient may be switched to a steroid sparing agent like methotrexate or tocilizumab (Actemra) as the prednisone is tapered slowly [7,10]. Monitoring always includes repeating laboratory values. If a patient has transient visual obscurations (graying or blacking out) or blurred vision due to choroidal nonperfusion or double vision, the prednisone dose should be at least 60 mg PO each morning with food [8,9]. If the ESR and CRP are very elevated, a significant thrombocytosis is present and/or the MRI shows extensive inflammation and/or the patient has already lost vision in one eye, the patient should be treated with high doses of IV steroids (methylprednisolone 250 mg q6 h) as an inpatient [8,9]. The characteristic severe visual damage is not reversible, but IV steroids usually prevent contralateral visual loss. Bilateral occipital lobe infarcts have been described. These patients can also have increased morbidity from stroke, myocardial infarctions, or aortic aneurysmal rupture if not treated [6,8,9].

## 3. Case 2

A 25-year-old woman with a past medical history of polysubstance abuse presents to the ED with a chief complaint of severe headaches that wake her from sleep and are present on awakening. She has tried NSAIDS without any improvement. She admits to alcohol, marijuana, and methamphetamine use and asks for Vicodin. Her physical examination is normal, and a non-contrast CT of the brain is normal. She is discharged with a limited supply of Vicodin and referred to outpatient neurology for migraine management. Her insurer is Medicaid, and she finds it difficult to visit a neurologist who will accept her insurance. She returns to the ED seven additional times with the same complaint. On her most recent visit, she complains of transient visual obscurations that gray out or black out her vision for seconds to minutes. She is again referred to Neurology and this time to Ophthalmology as well. Again, no one accepts her insurance and she presents to the ED for an eighth visit. On this visit, she complains of severe central visual loss bilaterally and on examination is unable to see more than the “big E” on the Snellen eye chart bilaterally. Her pupils are round but minimally reactive to light. No afferent pupillary defect (APD) is present. A fundoscopic exam is not obtained given that she is uncooperative (crying hysterically), there is no protocol for pupil dilation, and a non-mydriatic camera is unavailable. The ophthalmologist on call is slow to answer and the patient is admitted but unfortunately, the call group does not cover inpatients.

### What Would Have Been an Optimal Work Up in the Patient? Could the Visual Loss Have Been Prevented?

Women of childbearing age who are overweight are the population most at increased risk for idiopathic intracranial hypertension (high intracranial pressure with no specific cause) [14,15,16,17]. It can also occur in women of normal BMI as well as men [18,19,20]. Exposure to steroids, doxycycline, or other medications can trigger this disorder [16,18,19].

Early morning headaches should raise concern for increased intracranial pressure and/or an intracranial mass. The headaches often get worse when laying down (gravity dependent) and can be accompanied by transient visual loss when changing from lying to sitting or standing [18,21,22]. Increased intracranial pressure (ICP) is serious and must be addressed. Patients who complain of early morning headaches should always have their eyes examined for papilledema whether or not they have visual symptoms [18,21,22]. High Intracranial pressure (ICP) causes insidious visual field loss that begins in the periphery and is seldom noticed early on [15,18,21,23]. In some patients, diplopia also occurs due to the sixth cranial nerve being stretched across the petrous ridge [15,19]. 

The clinical history is critically important in this case too. Key symptoms are listed in Table 3. Patients should be asked about transient graying out or blacking out of vision especially when going from lying down or sitting to standing [15,19,21]. This patient ideally would have been referred to ophthalmology at the initial visit and had a non-mydriatic photo taken of the optic nerve. Rather than a non-contrast CT scan, an MRI/MRV of the brain would have been the imaging study of choice [18,19]. Venous sinus thrombosis can cause increased ICP [24]. While in the ED, a lumbar puncture should be performed in the lateral decubitus position to document opening pressure but also cells, glucose, and protein [19,21,25]. An elevated protein should prompt an MRI of the spine as a spinal tumor may be causing the increased ICP [19,25]. The differential diagnosis also includes indolent infectious diseases, such as tuberculosis and inflammatory entities including sarcoidosis [25].

This is a very treatable pathophysiology, especially if identified early. Patients are placed on acetazolamide—Diamox Sequels provide extended release—and can be dosed at 500 mg PO BID [18,19]. When caught early, vision is preserved. Weight loss alone may recommended in mild cases with minor symptoms and preserved visual function (vision, color, visual field, and mild papilledema). Once the process has caused central visual loss, the prognosis is guarded and admission for acetazolamide, and lumboperitoneal or ventriculoperitoneal shunting is the standard of care [15,18,19,26]. Venous sinus stenting is also an option in select patients. Optic nerve sheath fenestration is also an option but most helpful before the loss of central vision [19,21,25,27].

## 4. Case 3

A 40-year-old woman presents to the ED with neck pain and non-specific neurologic symptoms including numbness, tingling, and headaches. She denies any other symptoms. A non-contrast CT of her brain is performed, which is normal. Tele-neurology is consulted, but her symptoms do not fit the stroke protocol, so no recommendations are made. The patient is discharged without any specific instructions for follow-up.

Four weeks later she returns to the ED with bilateral visual loss. She first notices visual blurring several days prior to presentation. She denies any other neurologic symptoms, has no family history of vision problems, and is otherwise healthy on no medications. On examination she is unable to see anything on the eye chart but can appreciate light. Her pupils are round, reactive to light, and without an afferent pupillary defect. The anterior segment, IOP, and eye movements are normal. The ophthalmologist on call is contacted and recommends transfer to the university hospital 90 miles away. Tele-neurology is contacted, and they recommend a CT/CTA, which are both normal. They also recommend transfer to a university. A transfer is requested but all universities in the state were on diversion and refused transfer. Attempts to see the fundus with a direct ophthalmoscope are unsuccessful.

### What Would Have Been an Optimal Work Up in the Patient? Could the Visual Loss Have Been Prevented?

Devastating unilateral or bilateral visual loss can occur due to a wide variety of causes. The differential diagnosis includes compressive, infectious, inflammatory, toxic, vascular, neoplastic, or hereditary causes [28,29,30]. The initial evaluation in the ED can be very helpful in guiding therapy and preserving whatever vision is present. When a patient presents with visual blurring, the first step is to determine if the problem is in the retina or the optic nerve by taking a history and performing eye signs (i.e., vitals) including red desaturation, Amsler grid testing, and fundus photography [19,31]. The classic symptoms of retina vs optic nerve symptoms are presented in Table 4. Once it has been determined that it is an optic nerve issue, the age of the patient will guide the work-up even more than the appearance of the nerve.

**Figure 1:**
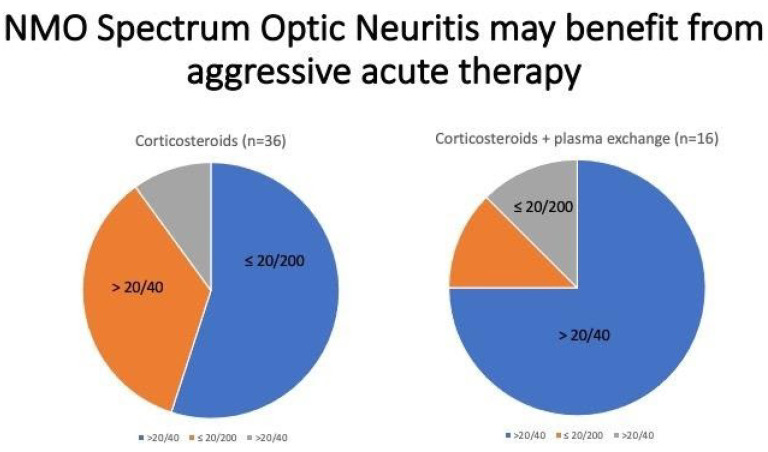
Graphical representation of treatment outcomes of Neuromyelitis optica spectrum optic neuritis with corticosteroids alone compared to treatment of NMO spectrum optic neuritis with combination of corticosteroids and plasma exchange [32].

Prior to the advent of MR imaging, vague neurological symptoms were difficult to evaluate. Both multiple sclerosis and neuromyelitis optica have characteristic findings on MRI and lumbar puncture [28,29,30]. Both are serious diseases that cause both visual and/or neurologic disabilities that can be permanent. However, treatment can be sight-saving as described in Figure 1. Table 5 presents the most common etiologies of bilateral vs unilateral visual loss. A non-contrast CT is an inadequate test for this population. If the testing is done prior to visual loss, the patient can be treated with IV steroids and referred for outpatient initiation of definitive therapy [31,32,33,34]. Distinguishing between MS-related optic neuritis and NMO-related optic neuritis is of prime importance because early initiation of effective immunosuppressive therapy is key to preventing relapses and permanent disability—see Table 6 [31,33].

## 5. Case 4

A 50-year-old man presents with acute onset of double vision. His eye vitals are otherwise normal. He has a past medical history significant for diabetes, hypertension, and hypercholesterolemia. He denies headache or eye pain. The ophthalmologist on call was unable to be reached. The tele-neurologist recommended a non-contrast CT/CTA, which was reported to be normal. No additional testing was done, and the patient was discharged and told to follow-up with an ophthalmologist. One week later, the patient is found down and arrives at the ED in an ambulance. The patient never regains consciousness and passes away from a ruptured aneurysm.

### What Would Have Been an Optimal Work Up in the Patient? Could This Have Been Prevented?

Managing double vision can prove equally as challenging as managing visual loss without an accurate ophthalmic examination. In a university-based ED setting, patients are typically seen in person by the ophthalmology residents on call, who are in turn supervised by a neuro-ophthalmologist. The neuro-ophthalmologist is then able to confirm a clinical diagnosis of a cranial nerve palsy or any other etiology of double vision. Depending on the diagnosis, the appropriate radiologic imaging protocol is followed and then interpreted by a neuro-radiologist. In the community-based ED setting, this stepwise evaluation and approach is not readily available. In this setting, a very helpful starting point is to take comprehensive external photos of the patient in the nine positions of gaze (i.e., straight ahead, up, down (with eyelids held up), left, right etc.). A list of the recommended diagnostic work up for common causes of double vision are presented in Table 7.

## 6. Case 5

A 58-year-old Caucasian man did a video visit with his primary care physician, in which he complained of severe pain in the distribution of his herpes zoster that had occurred years before. No vesicles were visible. He was placed on nonsteroidal anti-inflammatory during the day and Tylenol with codeine at bedtime. Despite receiving the Pfizer COVID vaccination seven months earlier, he presented to the ED with a fever, fatigue, muscle aches, sinus congestion, and a cough. COVID PCR testing was positive, but chest X-ray was normal. A comprehensive metabolic panel and complete blood count were normal. He was discharged to quarantine at home. Two days later, the patient returned to the ED with acute loss of vision in both eyes to 20/400, no relative afferent pupillary defect was present, and fundus photography in the ED with non-mydriatic camera was normal. Additional laboratory assessments that were found to be abnormal included elevated erythrocyte sedimentation rate (40), C-reactive protein (33), and D dimers (2000). Chest CT revealed ground glass changes consistent with COVID-19; pulse ox revealed diminished saturation of 88%. A non-contrast head CT was normal, but an MRI of the brain and orbits revealed a large occipital stroke. The patient was admitted for Decadron, anticoagulation, and supplemental oxygen. Access to the monoclonal antibody was denied. The inflammatory markers and D dimer normalized, and pulmonary function improved. The visual loss was permanent.

### What Would Have Been an Optimal Work Up in the Patient? Could Visual Loss Have Been Prevented?

COVID-19 (SARS-CoV-2) infections classically present with symptoms of fever, cough, fatigue, muscle aches, and neurologic alterations that result in loss of smell and taste [80,81,82]. The neurologic and ocular manifestations are less well known, and the understanding of optimal management is in evolution. It has been postulated, however, that live virus can potentially be found in the tear film [83,84,85,86]. Additionally, the virus can travel via ACE2 receptors through intact ocular epithelium and the endothelial lining of organs [84,87]. Ocular symptoms can be as mild as hemorrhagic conjunctivitis to as vision-threatening as retinal vascular occlusions and posterior ischemic optic neuropathy as listed in Table 8 [88,89]. Though the literature is limited, there have been several reported cases of the latter. In these cases, the typical presentation to the ED is with complaints of acute, painless, monocular, or binocular vision loss in the setting of a previous or recent diagnosis of COVID-19 (though this has also been reported in patients with a previous COVID-19 diagnosis > 3 months) [90]. Oftentimes, these patients have multiple chronic conditions that make them more susceptible to a more severe disease course. Positive patients with elevated inflammatory markers (IL-6, CRP, ESR, and fibrinogen) and d-dimer are at the highest risk for visual loss [91,92,93]. Thus, it is very important for the clinician to have a high index of suspicion for the patient that presents with elevated markers. Given that COVID-19 lowers the threshold for thrombotic complication, especially in the chronically ill, Decadron and anti-coagulation may prevent visual loss in patients with cytokine storm and hypercoagulability [89,94]. In certain cases, this may mean expedited complete visual recovery whereas in other instances, vision may improve spontaneously over time if at all [16].

## 7. Conclusions

Patients who present with acute visual loss, double vision, or other neuro-ophthalmic disorders often create a diagnostic and management challenge for the community hospital ED. Neuro-ophthalmology is outside of the comfort zone for most ED physicians, tele-neurologists, ophthalmologists, and radiologists. Gathering key historical information, performing eye vitals, and capturing fundus and/or gaze images helps to guide the work-up in the ED and the interface with tele-neurology and the on-call ophthalmologist. Failing to obtain the appropriate laboratory evaluations, imaging, and diagnostic interventions in the ED can result in permanent visual loss or death. Especially in communities where managed care complicates timely work-up on our neediest patients, the ED plays a central role in care for these patients. We hope that these clinical guidelines for neuro-ophthalmic patients can be integrated into EHR systems to help facilitate high-quality patient care in community EDs across the country.

## Figures and Tables

**Table 1:** Common ocular manifestation of giant cell arteritis and appropriate timeline for consultation and management in the emergency department [5,6].

Symptom	Treatment Dosage
Eye pain	Prednisone 60 mg PO each am Eye exam within 24 h *
Transient visual obscurations (TVOs)	Prednisone 60 mg PO each am Eye exam within 24 h *
Blurred vision or mild visual acuity (VA) change	Prednisone 60 mg PO each am Eye exam within 24 h *
Devastating visual loss (anterior ischemic optic neuropathy, posterior ischemic optic neuropathy, cilioretinal artery occlusion, central retinal artery occlusion) resulting in VA < 20/400, no light perception not uncommon. Occipital lobe infarctions have also been described.	Admit for IV corticosteroids—start first dose in the ED. Ophthalmology consult as inpatient.
Double vision due to cranial nerve (CN) III, IV, VI involvement	Discharge on prednisone 60 mg PO each am Ophthalmology exam within 24 h *

* If the ESR, CRP, CBC, A-SSA are significantly elevated—Admit for IV steroids. Always start corticosteroids in ED there can be a significant delay in getting dosed on the ward.

**Table 2:** Sensitivity of Lab Testing for GCA Patients by percentage.

Lab Value	Sensitivity
ESR	76–86% (Parikh et al., 2006) [11]
CRP	97.5% (Parikh et al., 2006) [11]
ESR and CRP	99% (Parikh et al., 2006) [11]
Platelets	71.2% (Franzco et al., 2021) [12]
Acute serum amyloid A (A-SAA)	97% (Franzco et al., 2021) [12]
Normocytic normochromic anemia	20–50% (Franzco et al., 2021) [12]
PLT > 634 k, ESR > 90, CRP > 115	All had positive TA BX (Weis et al., 2021) [13]
PLT < 224, CRP < 2, ESR < 9	All had negative TA BX (Weis et al., 2021) [13]

ESR—erythrocyte sedimentation rate, CRP—C-Reactive protein, PLT—platelet.

**Table 3:** Clinical manifestations of increased ICP and optimal management in the ED setting [15,19,21].

Symptoms	Diagnostic Work-Up
Headache often worse in the am or wakes patient from sleep	Eye Vitals: vision, intraocular pressure (IOP), red desaturation, amsler grid, confrontation visual field, motility, and fundus photo (optic nerve and central retina)
Transient graying out or blacking out of vision	MRI/MRV
Double vision (CN VI dysfunction)	Followed by lumbar puncture (LP) in lateral decubitus position for opening pressure, cell, protein, and glucose
Visual field defects	If central visual acuity is affected, especially if papilledema looks ischemic (cotton wool spots), admit
Decreased vision	-

**Table 4:** Retina versus optic nerve etiologies of classic symptoms [31,32,33].

Retina Symptoms and Signs	Optic Nerve Symptoms and Signs
Retina Classic Symptoms: Flashing lights Floaters Shadow that progresses from the periphery	Optic Nerve Classic Symptoms: Graying out or blacking out Eye pain or ache Eye pain worse on eye movement Woke up with visual loss
Amsler grid: wavy lines Red color is normal Ultrasound: vitreous opacification can be blood or infection. Retinal detachment may be visible. (need to have gain turned all of the way up)	Amsler grid: Absent lines or regions Red desaturation Ultrasound: can detect severe disc edema
Fundus photo of Retina can show: Vitreous hemorrhage Diabetic retinopathy Macular degeneration with hemorrhage Macular fluid (easier to see on OCT) Central retinal artery occlusion Branch retinal artery occlusion Central retinal vein occlusion	Fundus photo of optic nerve can be: - normal (posterior ischemic optic neuropathy, retrobulbar neuritis) - pale (compression from tumor, toxic from alcohol or a medicine, infection like syphilis, tuberculosis, lyme disease, etc., the process has been going on for several weeks or more) - swollen (anterior ischemic optic neuropathy, papillitis, optic neuritis, papilledema, central vein occlusion) - cupped from end-stage glaucoma

**Table 5:** Differential of optic neuropathies categorized by age and appropriate testing and work up in the ED setting [28,29,30].

Age	Unilateral or Bilateral Visual Loss	Unilateral Visual Loss	Bilateral Visual Loss
AGE > 60	-	Non-arteritic ischemic optic neuropathy Giance cell arteritisCompressive (orbital mass)	Compressive (parasellar mass) Toxic (Alcohol, ethambutol) Infectious (TB, syphilis, Lyme)
AGE < 60	Optic Neuritis Neruomyelitis Optica Spectrum Disorder (NMOSD) Lupus Saracoidosis	-	Compressive (parasellar mass) Toxic (alchol, ethambutol) Infectious (TB, syphilis, lyme)
**Unilateral Visual Loss Testing in ED:**		**Bilateral Visual Loss Testing in ED**	
Eye vitals, fundus photo CBC, ESR, CRP, RPR, Quantiferon MRI of brain and orbit with/without GAD		Eye vitals including fundus photo ANA, ACE, RPR, Quantiferon Anti-aquaporin-4 antibody (AQP4) MRI of brain and orbit with/without GAD Lumbar puncture	

**Table 6:** Signs and symptoms differentiating Multiple Sclerosis Optic Neuritis and NMO-related Optic Neuritis [25,26,29,30].

Presenting Symptoms	Signs
MS-Related Optic Neuritis	NMO-Related Optic Neuritis
Unilateral visual loss better than 20/100 improves in 6–8 weeks	Unilateral or bilateral visual loss worse than 20/100 and often permanent
No biomarker	anti-AQP4 biomarker
Can be retrobulbar or with disc edema Eye pain worse on eye movement More common in women than men	Can be retrobulbar or with disc edema Eye pain worse on eye movement More common in women than men
Short regions of enhancement on MRI orbits	Long regions of optic nerve enhancement that extend to the chiasm and may be bilateral. Longitudinally extensive transverse myelitis (LETM) that spans 3 or more vertebral segments
Periventricular plaques on MRI brain	Subcortical and deep white matter lesions on T2-weighted or fluid-attenuated inversion recovery sequences. Diencephalic lesions around the third ventricle, thalamus, hypothalamus, and midbrain. Dorsal brainstem adjacent to the fourth ventricle also reported that it causes intractable hiccups, nausea, and vomiting. Nystagmus, dysarthria, dysphagia, ataxia and ophthalmoplegia (multiple cranial nerves causing dysmotility) can also occur. Can also present with antidiuretic hormone secretion, narcolepsy, hypothermia, hypotension, hypersomnia, obesity, hypothyroidism, hyperprolactinemia, amenorrhea, galactorrhea, and behavioral changes.
LP: leukocytosis	LP: oligoclonal bands

**Table 7:** Work up and management of binocular diplopia in the ED setting.

Cranial Nerve Palsy	Diagnostic Work-Up
Complete CN III with pupil involvement [35,36,37,38] Eyelid drooping (ptosis), eye position is down and out. Pupil is larger than contralateral pupil	Aneurysm until proven otherwise. If Computed tomography angiography (CTA) is normal, the standard of care is to transfer to hospital with interventional radiology to perform angiogram
Incomplete CN III [35,36,37,38] Limitation of up gaze with ptosis and/or limitation of downgaze and contralateral gaze (adduction).	CTA and close follow-up for progression HBA1c CBC, ESR, CRP if patient > 60 years old
CN IV [35,36,37,38,39,40] Vertical deviation worse with tilt of head on side of higher eye	MRI with and without gadolinium HBA1c CBC, ESR, CRP if patient > 60 years old
CN VI [35,38,40] Limitation of lateral gaze	MRI with and without Gad HBA1c LP for opening pressure, cells, protein, glucose
Thyroid eye disease [41,42,43] Vertical deviation in primary gaze Limitation of up gaze Limitation of lateral gaze Esotropia in primary gaze Proptosis (bulging of eyes) Eyelid retraction (appearance of stare)	CT scan of orbits (include corneal views) TSH, free T3 and T4, Thyroid stimulating immunoglobulin
Myasthenia Gravis [43,44,45,46,47] Double vision worse at end of day or with reading Any deviation pattern possible Associated with drooping of eyelid (ptosis)	CT scan of chest looking for thymoma Acetylcholine binding, blocking and modulating antibodies and anti-musk
Orbital Fracture [48,49,50] Recent trauma to orbit with limitation of up gaze (or less commonly lateral and downgaze)	CT scan of orbit to look for blow out fracture
Pituitary apoplexy [51,52,53,54,55,56,57,58,59,60,61] Any or all cranial nerves can be involved	Non-contrast CT will typically identify the parasellar hemorrhage and necrosis. IV corticosteroids should be initiated and urgent transfer to a hospital with Neurosurgery.
Orbital Apex Syndrome [62,63,64,65,66,67,68,69] Any or all cranial nerves can be involved Orbital pain and visual field/visual loss also typically present	MRI of orbits/brain with and without GAD Differential includes mucor, aspergillosis, non-specific inflammatory process, sarcoidosis, and lymphoma. ENT and ophthalmology consultation. Admit for IV corticosteroids and anti-fungal medications.
Tolosa Hunt [70,71,72,73,74] Any or all cranial nerves can be involved Orbital pain characteristic	MRI of brain with and without Gad To detect enhancement of cavernous sinus Differential includes mucor, aspergillosis, non-specific inflammatory process, sarcoidosis, lymphoma, and cavernous sinus thrombosis. Admit for IV corticosteroids.
Cavernous sinus fistula/Sinus Thrombosis [75,76,77,78,79,80,81,82] Any or all cranial nerves can be involved Conjunctival injection and chemosis typical Proptosis (eye bulging may be present) Tinnitus (whooshing in the ears)	CT/CTA will show superior ophthalmic vein enlargement, extraocular muscle swelling and convexity to the normally concave wall of the cavernous sinus. Initiate transfer to hospital with interventional

**Table 8:** Ophthalmic and neurologic manifestations of COVID-19 infection [88,89].

Ocular Manifestations of COVID-19
Conjunctivitis (hemorrhagic)
Scleritis
Retinal infarcts
Orbital infiltration
Central retinal vein occlusion; branch retinal vein occlusion
Central retinal artery occlusion; branch retinal artery occlusion
Anterior visual pathway strokes resulting in visual loss
Posterior visual pathway strokes resulting in visual loss
Cerebral venous thrombosis with papilledema
Cavernous sinus thrombosis with diplopia
